# The Value of a Predictive Model Based on Multimodal Ultrasound Imaging Biomarkers Combined with Clinical Features in the Diagnosis of Thyroid Nodules

**DOI:** 10.2174/0115734056458403260609191638

**Published:** 2026-06-22

**Authors:** Huajie Ding, Lei Na, Meiling Hao, Wanlou Chen, Zhen Zhang

**Affiliations:** 1 Department of Ultrasound, Affiliated Hospital of Chengde Medical University, Chengde 067000, China;; 2 Department of Emergency, Affiliated Hospital of Chengde Medical University, Chengde 067000, China;; 3 Department of Pathology, Affiliated Hospital of Chengde Medical University, Chengde 067000, China;; 4 Department of Ultrasound, Liaoning Cancer Hospital & Institute, Shenyang, 110000, China

**Keywords:** Machine learning, Multimodal ultrasound-based imaging omics, SMI, Thyroid nodules, Diagnostic value

## Abstract

**Introduction:**

This study aimed to develop and validate diagnostic models for distinguishing benign and malignant thyroid nodules.

**Methods:**

Between January 2020 and June 2024, 735 patients were retrospectively selected for surgery or needle biopsy at our hospital. This cohort was divided into a training cohort (n=514) and a validation cohort (n=221) in a ratio of 7:3. All models were validated 10 times, and a receiver operating characteristic curve (ROC curve) was generated. Furthermore, a nomogram prediction model was constructed using the independent risk factors from Model 3, and its validity was verified through ROC curves, calibration curves, and clinical decision curves.

**Results:**

The AUC of Model 1 in the training cohort was 0.889, 0.913, 0.914, 0.914, 0.928, and 0.910, respectively. The AUC of Model 2 in the training cohort was 0.950, 0.957, 0.936, 0.946, 0.963, and 0.921, respectively. The AUC of Model 3 in the training cohort was 0.985, 0.991, 0.969, 0.949, 0.866, and 0.986, respectively. The Model 3, which was constructed based on the optimal classifier LDA, had the highest predictive value. Moreover, the clinical decision curve also indicated that it offers the greatest net benefit to patients.

**Discussion:**

The integration of clinical features with multimodal ultrasound imageomics significantly improved diagnostic accuracy. The combined model showed enhanced sensitivity and specificity, potentially reducing unnecessary invasive procedures.

**Conclusion:**

This study developed a non-invasive preoperative diagnostic method for thyroid nodules using multimodal ultrasound histology and clinical models, and its nomogram was also convenient for clinical application.

## 1 INTRODUCTION

Thyroid nodules are lumps formed by the localized abnormal growth of thyroid cells, and their global incidence is increasing, with approximately 10% to 15% progressing to malignant tumors [1]. It is estimated that by 2030, thyroid cancer will become the second most common cancer among women, with an incidence rate exceeding that of other cancers [2, 3]. Therefore, early diagnosis of thyroid nodules and accurate differentiation of benign and malignant nodules are of great importance for clinicians to provide active management guidance [4].

Although conventional ultrasound is the preferred method for examining thyroid nodules, there are multiple versions of the Thyroid Imaging Reporting and Data System (TIRADS), and there is a lack of unified understanding [5, 6]. This leads to certain limitations in the diagnosis of the disease [7]. Ultrasound elastography is widely used in the diagnosis of diseases affecting various organs [8, 9]. However, in the specific diagnosis of thyroid nodules, false positives may occur, thereby affecting the identification of nodules [10] Superb microvascular imaging (SMI) is a new type of ultrasound technology that can assess the tumor microvascular system [11]. Although it can partially compensate for the shortcomings of conventional ultrasound, it still has limitations in standardization and the physical limits of the technology itself [12]. Currently, ultrasound-guided biopsy is still the second-best standard for preoperative diagnosis, but 10%~30% of nodules cannot be confirmed by pathological diagnosis [13]. Therefore, the current diagnostic technical level of thyroid nodules still needs to be further improved, especially in non-invasive diagnostic techniques and levels.

Imageomics-based analysis is an important research method involving computer engineering to promote precision medicine [14]. Current studies have shown that Imageomics-based analysis can not only be used to evaluate benign and malignant nodules, but also to determine the presence of lymph node metastasis [15, 16]. In recent years, imageomics-based analysis has demonstrated great potential in differentiating benign and malignant thyroid nodules. By extracting features such as the gray-level co-occurrence matrix from ultrasound images using texture analysis methods and combining them with a support vector machine classifier, a performance of AUC 0.85 was achieved. However, this study was conducted in a single center, based solely on the intratumoral region, and did not incorporate clinical features [17]. It was further expanded to a multi-center cohort, exploring the integration of peritumoral regions and manual features with deep learning features, and comparing the evaluations of radiologists. The results showed that the fusion model achieved an AUC of 0.92, but still had issues such as high feature dimensions and insufficient interpretability [18]. However, few reports have combined imaging omics with multimodal imaging using traditional ultrasound, SMI, and elasticity imaging to diagnose thyroid nodules. Therefore, this study aims to establish a multimodal ultrasound imaging omics model based on 2D ultrasound images (longitudinal and transverse) and SMI, analyze the diagnostic value of 6 classifier models, incorporate independent risk factors of clinical ultrasound into the diagnostic model, establish a combined diagnostic model, and explore the diagnostic value of different diagnostic models for thyroid nodules. We developed a dedicated nomogram for joint diagnosis to assist in selecting appropriate clinical treatment plans.

## 2 MATERIALS AND METHODS

### 2.1 Study Participants

In this study, 735 thyroid nodules from 735 patients treated between January 2020 and June 2024 were included. All patients underwent 2D ultrasound mapping (longitudinal and transverse sections), SMI, and SE, and the clinical data met the inclusion criteria. There were no issues with omissions or absences. The cohort was restricted to a 7:3 ratio, with training and validation cohorts of 514 and 221 patients, respectively. Based on the results of needle aspiration biopsy or pathological diagnosis, the research subjects were divided into two groups: the benign group and the malignant group. In the training set, there were 242 cases in the benign group and 272 cases in the malignant group. The number of cases in the benign group and the malignant group in the validation set was 104 and 117, respectively.

Sample size selection: We conducted sample size estimation using PASS. 2025. Based on the expected effect size as stated in the literature [15, 16], we set the significance level at 0.05 and the required power at 80%. The calculated required sample size is approximately 700, which is very close to the 735 sample numbers we ultimately included in this study. Additionally, dividing the research subjects into a training set and a validation set in a 7:3 ratio is a very mature method in machine learning and statistical modeling. In our study, the training set of 514 patients and the validation set of 221 patients were both large enough to effectively train and validate the diagnostic model.

The inclusion criteria were as follows: (1) patients with pathologically diagnosed thyroid nodules after needle biopsy or surgery; (2) patients for whom complete preoperative ultrasound diagnostic images could be obtained and preserved in the Digital Imaging and Communications in Medicine (DICOM) format; (3) patients who did not receive a series of anticancer treatments before surgery.

The exclusion criteria were as follows: (1) patients whose pathological diagnoses could not be specified; (2) patients for whom imaging and nodule data were incomplete for various reasons, such as anatomy, location, or machine use; (3) patients for whom clinical or pathological data were incomplete. This study was approved by the Ethics Committee of the Affiliated Hospital of Chengde Medical University (approval number: CFYFLL2023313). Informed consent was obtained from all patients for the use of their clinical data and images.

### 2.2 Instrument, Equipment, and Operation Methods

#### 2.2.1 Clinical Characteristics and Ultrasound Images

The clinical data and basic ultrasound image characteristics of patients in the training and validation cohorts were analyzed. Clinical characteristics were referred to the AJCC TNM staging guidelines for differentiated thyroid cancer [19]. Specifically, the patients were assigned to two groups according to age (≥55 years) [20] and sex (male or female). Two senior doctors experienced in thyroid nodule ultrasound diagnosis guided the procedure and analyzed the image data, which included: (1) target nodule size (the upper and lower diameters, the front and back diameters and the left and right diameters of the nodule, and selection of the maximum value for recording [≤1 cm]); (2) nodular echogenicity, including mainly low echo or very low echo, medium and high ech and mixed echo. (3) nodule boundary: clear or unclear; (4) number of nodules (regular or irregular); (5) A/T representative of the ratio of both the longitudinal diameter and the transverse diameter of the largest section of the nodule (≥1 cm); (6) nodule calcification, in which microcalcification was indicated by a strong echo from suspected calcification that did not exceed 1 mm; (7) demarcation of the nodule and capsule, whether there was a clear demarcation between the nodule and capsule through the vertical (transverse) section (clear or unclear); (8) multifocal, classified as multifocal (≥2 internal carcinoma foci) or not multifocal (based on the preoperative ultrasound examination results). (9) SMI patter [21], evaluated according to the observed blood supply of the nodules.

#### 2.2.2 Acquisition of Multimodal Ultrasound Images

GE LOGIQ E10,E11 ultrasonic diagnostic instrument with a 4-20 MHz adjustable broadband line array probe, as well as SMI and SE technologies, was used. An ultrasound examination should be conducted within 24 hours after the patient is admitted to the hospital. The patient was placed in the supine position, and the neck was fully exposed. Two experienced senior ultrasound doctors simultaneously conducted the thyroid examination on the patient and determined the location of the target nodule. Furthermore, the largest representative longitudinal and transverse-section nodule images were obtained to improve patient basic and imaging data. For the SMI mode, after determining the target nodule, the largest longitudinal-section image was obtained. The SMI mode was initiated, and a stable blood flow map was selected. In the SE mode, the target nodules were adjusted to the appropriate central image position. Normal tissues and target nodules were identified, and patients were required to maintain a steady breathing state. The probe and neck were vertical, and a small vibration state was maintained. After the pressure quality control index was fully green, a stable 2-3 s frozen image was used. The image was saved, and an elastography map was obtained. Two experienced senior ultrasound doctors simultaneously conducted the thyroid examination and image acquisition for the patient. Subsequently, the two physicians separately carried out the collection of data parameters.

#### 2.2.3 Region of Interest Segmentation for Multimodal Ultrasound Images

The most typical and representative DICOM ultrasound images were exported from the physician's Picture Archiving and Communication System workstation. As both SMI and SE images contain color layers, the images were subsequently normalized and pretreated to facilitate texture analysis. After pretreatment, the four modal patterns of longitudinal section, transverse section, SMI, and SE were presented as gray-scale images of the same mode, which was conducive to feature extraction and texture analysis. Two experienced ultrasound doctors (both with over 10 years of experience in thyroid ultrasound examinations) separately used the ITK-SNAP software (version 4.0.1) to manually segment all 734 nodules. Both doctors were unaware of the pathological diagnosis results, clinical data, and all other information. To evaluate the repeatability between observers, the same observer re-segmented 50 randomly selected nodules two weeks later. The intraclass correlation coefficient (ICC) calculated using the two-way random effects model was used to evaluate the repeatability between observers and the repeatability within observers, which was used to measure the absolute consistency. If the ICC values for both the repeatability between observers and the repeatability within observers were greater than 0.75, then the image feature extraction results were considered to be in compliance with the requirements. The following shows the range of the inter-observer correlation coefficient (ICC) for each imaging modality: Two-dimensional ultrasound (longitudinal section): ICC range is 0.81-0.94; Two-dimensional ultrasound (cross-sectional image): ICC range is 0.79-0.92; SMI: ICC range is 0.76-0.89; SE: ICC range is 0.74-0.8; In addition to the ICC, we also calculated the Dice similarity coefficient (DSC) to evaluate the overlap degree between the segmentation results of the same group of 50 nodules by the two readers. The average DSC was 0.89±0.05 (range: 0.82-0.95), indicating excellent consistency in ROI depiction. For cases with significant discrepancies (DSC of key features less than 0.80 or ICC less than 0.75), a senior ultrasound doctor with over 15 years of experience would review the case and facilitate the consistent segmentation of opinions. The consistent segmentation results were used as the final reference standard for feature extraction.

#### 2.2.4 Characteristic Extraction and Selection, and Multimodal Ultrasound-based Imaging Omics Model Establishment

Multimodal ultrasound imaging features were extracted from four image types (2D ultrasound longitudinal sections, 2D ultrasound transverse sections, SMI, and SE) using FAE Pro 0.3.6 open-source software. For each modality, 1317 quantitative features were obtained, resulting in a total of 5268 features per nodule. These features encompassed: (i) morphological features (ROI size, shape, surface area, volume, surface-to-volume ratio); (ii) first-order statistics derived from the global grey-level histogram (mean, maximum, minimum, variance, percentiles, skewness, kurtosis); and (iii) second-order texture features including the grey-level co-occurrence matrix (GLCM), grey-level run-length matrix (GLRLM), grey-level size zone matrix (GLSZM), neighbouring grey tone difference matrix (NGTDM), and grey-level dependence matrix (GLDM). All features were extracted from the original ultrasound images as well as from filtered and derived images. Prior to analysis, feature values were normalised using Z-score transformation (z=x−μσz=σx−μ , where μ and σ are the mean and standard deviation computed from the training cohort) to ensure comparability across different scales and to prevent data leakage. To guarantee the reproducibility and stability of feature extraction, two experienced sonographers independently delineated the ROIs (see section 2.2.3). The ICC was calculated using a two-way random effects model for absolute agreement. Only features with an ICC>0.75 were retained for subsequent analysis. Among the 5268 extracted features, 4875 satisfied this criterion.

Feature selection was performed exclusively on the training cohort (n=514) using a rigorous multi-step strategy to reduce dimensionality, avoid overfitting, and prevent data leakage. The entire selection pipeline followed the standard imageomics workflow: stability filtering, variance filtering, univariate association testing, multivariate selection with embedded cross-validation, and multicollinearity removal. The specific steps were as follows:

Step 1: Low-variance filtering. Features with extremely low variance (variance threshold<0.01) were removed, as they provide little discriminatory information.

Step 2: Univariate association filtering. The association between each remaining feature and the pathological outcome was evaluated using the Kruskal–Wallis test for continuous features and the chi-square test for categorical features; only features with *P*<0.05 were retained.

Step 3: Multivariate feature selection with cross-validation. To avoid overfitting and determine the optimal feature subset, the entire selection process from Step 3 onward was embedded within a 10-fold cross-validation loop on the training cohort. Each iteration is carried out independently, meaning that the features are selected solely based on the training set, and the performance of the model is evaluated on the corresponding validation set.Three complementary feature selection methods were applied in parallel to the training folds: (1) LASSO regression with 10-fold cross-validation: This method minimises the negative log-likelihood with an L1 penalty λ∑|βj |, shrinking some coefficients exactly to zero, thus performing intrinsic feature selection. The optimal λ was chosen as the one yielding the minimum cross-validated deviance within each iteration. (2) Recursive feature elimination (RFE) using a random forest classifier: Features were ranked by their importance (mean decrease in Gini impurity), and the least important feature was iteratively removed until the desired number of features was reached. The optimal number of features was determined by cross-validation. (3) Minimum redundancy maximum relevance (mRMR): This algorithm selects features that have maximal mutual information with the outcome while minimising mutual information among themselves, thereby reducing redundancy. After applying these three methods, we retained features that were selected by at least two of the three methods in each iteration. The final set of features for each diagnostic model was chosen as the one that maximised the average cross-validated AUC across all 10×10 iterations.

Step 4: Multicollinearity removal. For the final feature set obtained above, pairwise Pearson correlation coefficients were calculated:


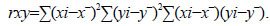


If any pair exhibited |r|>0.8 (indicating high collinearity), the feature with the weaker univariate association (based on the Kruskal–Wallis or chi-square test) was discarded. This step ensures that the final model is not distorted by redundant information.

To avoid data leakage and determine the optimal number of features, the entire feature selection pipeline was embedded within a 10-fold cross-validation loop repeated 10 times on the training cohort. In each iteration, features were selected using only the training folds, and the model performance AUC was evaluated on the corresponding validation fold. The final set of features for each diagnostic model was chosen as the one that maximised the average cross-validated AUC across all iterations. For the multimodal imaging omics model (Model 2), the procedure described above yielded 17 key features. For the combined model (Model 3), the same feature selection process was applied to a dataset that included both the imaging features and the independent clinical-ultrasound risk factors identified in Model 1 (age< 55 years [20], nodule irregularity, low/very low echogenicity, microcalcification, and absence of multifocality). This resulted in 20 features (17 imaging features and 3 clinical features) that were used to construct the final combined model.

After feature selection, six classifiers were trained on the selected features: support vector machine (SVM), linear discriminant analysis (LDA), logistic regression (LR), LASSO (used here as a classifier), decision tree (DT), and naive Bayes (NB). Model performance was evaluated in both the training and validation cohorts using AUC, sensitivity, specificity, positive predictive value, negative predictive value, and accuracy. All steps were implemented with FAE Pro 0.3.6 and in-house R scripts to ensure full reproducibility. The flowchart is in Fig. (**1**).

#### 2.2.5 Diagnostic Model Establishment and Evaluation

This study included three models. The clinical diagnostic model (Model 1) was based on the univariate analysis of the clinical and ultrasound characteristics of patients in the training cohort. Factors with significant differences (*P*<0.05) were selected for multivariate logistic regression analysis. Model 1 was established, and the AUC, sensitivity, specificity, positive predictive value, negative predictive value, and accuracy were assessed. The multimodal ultrasound-based imaging omics model (Model 2) was established after further normalization, dimension reduction, and screening. The calculation of the AUC and other evaluation parameters was performed using this method, based on the characteristics of multimodal ultrasound imaging omics, to assess their application efficiency. In the combined diagnostic model (Model 3), the best comprehensive classifier was selected from the second validation set, and the imaging omics features were combined with the multifactor analysis results of clinical ultrasound risk factors to generate new features. Model 3 was established after feature screening and dimension reduction. The final combined model (Model 3) is presented as a logistic regression formula based on the LDA classifier. The linear predictor (LP) is calculated as: LP=β_0_+β_1_·X_1_+β_2_·X_2_+...+β_20_·X_20_. where X_1_ to X_20_ are the selected features (normalized using the Z-scores derived from the training set), and β_0_ to β_20_ are the corresponding coefficients. The predicted probability of malignancy is then obtained by: *P*=1/(1+e^{-LP}). The complete list of features, their definitions, and the corresponding coefficients are provided in Appendix Table **A1**. For example, the top five contributing features were: SMI_texture_entropy (β=0.821), 2D_longitudinal_gray_mean (β=0.763), SE_skewness (β=0.708), microcalcification (β=0.612), and age≥55 years (β=0.587). The diagnostic efficiency of the AUC was calculated. The optimal diagnostic model for thyroid nodules was selected, and its value in clinical experiments was discussed.

#### 2.2.6 Generation of the Nomogram and Clinical Decision Curve of Model 3

The output from the best combination of diagnostic efficacy in Model 3 was used to obtain the imaging omics scores for weighted linearity. Clinical ultrasonography-independent risk factors were added to establish the nomogram. The calibration and diagnostic effects were evaluated using the consistency index, ROC curve, and calibration curve. The corresponding net benefit was obtained based on the threshold calculation results, and a clinical decision curve was constructed to further evaluate the clinical value of the diagnostic model.

### 2.3 Statistical Analysis

Statistical analysis was performed using FAE, SPSS, and R language software. The measurement data were expressed as the means ± standard deviations and analyzed *via* t tests or U tests. A two-sided test was used for between-group comparisons of count data. Independent risk factors in Model 1 were screened using univariate and multivariate logistic regression analyses. Intergroup correlation coefficients were used to evaluate whether the characteristics extracted using multimodal ultrasound imaging omics had good reliability and repeatability. The diagnostic performance of the evaluation model was analyzed based on the AUC, sensitivity, specificity, positive predictive value, negative predictive value, and accuracy. Receiver operating characteristic (ROC) curves for all diagnostic models were analyzed using the Delong test. The nomogram, calibration curve, and clinical decision curve of Model 3 were generated using the R language platform to evaluate its value in clinical practice. The comparison of AUC between the models was conducted using the Durbin test, and the Bonferroni correction method was applied for multiple comparisons. The *P*-values of all univariate tests (Kruskal-Wallis test, chi-square test) were corrected using the Bonferroni method, with the adjusted significance level being *P*<0.05/number of features. Additionally, the *P*-values reported in the results section are all the corrected values. Statistical significance was set at *P*<0.05.

## 3 RESULTS

### 3.1 Baseline Data, Clinical Manifestations, and Basic Ultrasound Features

A total of 735 patients were included in this study, and they were divided into the training group (n=514) and the validation group (n=221) in a ratio of 7:3. There was no difference in baseline data, such as gender, BMI, smoking history, drinking history, and membrane boundary, between the training group and the validation group (*P*>0.05) (Table **1**). There were significant differences between the two groups in terms of nodule size, echo characteristics, boundary, shape, aspect ratio, microcalcification, and multiple nodules (*P*<0.05) (Table **2**).

### 3.2 Establishing Model 1: Clinical Characteristics Combined with the Conventional Ultrasound Model

Multivariate binary logistic regression analysis revealed statistically significant differences in age (55 years; *P*=0.011), nodular irregularities (*P*=0.000), low or very low echogenicity (*P*<0.001), microcalcifications (*P*=0.047), and absence of multifocality (*P*=0.028) between the malignant and benign groups (Table **3**). Based on the aforementioned features, clinical diagnostic models were established using six classifiers: SVM, LDA, LR, LASSO, DT, and NB. In the training cohort, the AUC values for these six classifiers were 0.889, 0.913, 0.914, 0.914, 0.928, and 0.910, respectively. In the validation cohort, the AUC values were 0.858, 0.908, 0.907, 0.907, 0.891, and 0.908. In the validation cohort, the sensitivity values were 0.936, 0.848, 0.877, 0.877, 0.892, and 0.830; Specificity values were 0.771, 0.870, 0.855, 0.855, 0.798, and 0.867; and accuracy rates were 0.859, 0.859, 0.867, 0.867, 0.848, and 0.848 (Table **4**).

### 3.3 Establishing Model 2: Imaging Imageomics Model Based on Multimodal Ultrasound

A multimodal imaging platform was constructed based on B-mode ultrasound, strain elastography imaging and super microvascular imaging. A large number of quantitative features were extracted from the above images using the imaging biomarker method, and were rigorously screened for quality control: the intraclass correlation coefficient (ICC) of 4875 features was greater than 0.75, and 4183 features had an interclass ICC greater than 0.75. These results indicated that the diagnostic findings of the two sonographers were not considerably different; thus, feature extraction was stable and reliable (Fig. **2**). We eliminated 692 features with ICCs<0.75, whereas the remaining features were further normalized to reduce the possibility of overfitting. Ten repeated tests were performed to determine 17 key features from which a diagnostic model was established. In the training cohort, the AUCs of the different models were 0.950, 0.957, 0.936, 0.946, 0.963, and 0.921 for SVM, LDA, LR, LASSO, DT, and NB, respectively. The AUCs for the validation cohort were 0.933, 0.951, 0.930, 0.941, 0.929, and 0.918. The sensitivity in the validation cohort was 0.621, 0.729, 0.629, 0.657, 0.914, 0.764. The specificity in the validation cohort was 0.949, 0.937, 0.920, 0.922, 0.927, 0.876; The accuracy in the validation cohort was 0.866, 0.884, 0.846, 0.855, 0.924, 0.848. Among them, the LDA classifier demonstrated relatively strong performance in terms of AUC, specificity, and accuracy (Table **5**). Moreover, as a linear model, it had good interpretability, so it was selected as the final classifier. A comparison of the ROC curves for the six classifiers is shown in Fig. (**3**). The multimodal ultrasound image is shown in Fig. (**4**).

### 3.4 Construction of Model 3: Multimodal Ultrasound Imaging Biomarker Combined with Conventional Clinical Diagnostic Model

Thyroid nodules of patients with clinical and ultrasound-based risk factors (age<55 years, nodule irregularity, low or very low echogenicity, microcalcification, and lack of multifocality) were combined with the extracted multimodal ultrasound imaging features to generate a new feature set that was subsequently normalized. Thereafter, Pearson’s correlation was used for feature dimension reduction, and the REF classifier was used to screen typical features to achieve better data classification. Based on a comparison of the diagnostic efficiencies of Models 1 and 2, the LDA classifier with the best comprehensive performance was selected to establish Model 3. To determine the hyperparameters of the diagnostic model (such as the number of features), the characteristics of the training cohort were verified 10 times. Twenty key features were selected, including 17 imaging features and 3 clinical features (Fig. **5**). The best AUC results were reported. The AUC, sensitivity, specificity, positive predictive value, negative predictive value, and accuracy of the trained cohort were 0.991, 0.961, 0.949, 0.866, 0.986, and 0.952, respectively. Regarding the validation cohort, the AUC, sensitivity, specificity, positive predictive value, negative predictive value, and accuracy were 0.985, 0.957, 0.934, 0.842, 0.985, and 0.940, respectively. Distribution results were obtained by combining clinical ultrasound features with multimodal ultrasound-based imaging omics features (Fig. **6**). This figure allowed for the determination of consistency between thyroid nodule diagnosis and the pathological findings, demonstrating that the model had a good diagnostic efficiency.

### 3.5 Diagnostic Value of the Different Models

The sensitivity, specificity, predictive value, and accuracy of the different diagnostic models were summarized in Table **6**. Model 3 showed the best efficacy in both the training and validation cohorts, with AUCs of 0.991 (95% confidence interval [CI]: 0.987-0.994) and 0.985 (95%CI: 0.974-0.996), respectively. In the validation cohort, the AUCs of Models 1 (0.908[95%CI: 0.871-0.924]) and 2 (0.951[95%CI: 0.931-0.970]) were lower than those of Model 3 (*P*=0.00036; *P*=0.0166). In the validation cohorts, The sensitivity of the diagnostic models 1, 2 and 3 were 0.848, 0.729, 0.957 respectively, Showed that model 3 had significantly improved efficacy in detecting thyroid nodules, Low sensitivity of models 1 and 2 could lead to missed diagnosis of nodules; The specificity of models 1, 2 and 3 were 0.870, 0.937, 0.934, Model 2 and 3 had a slightly higher diagnostic efficacy for malignant thyroid nodules; The positive predictive values of models 1, 2 and 3 were 0.880, 0.814, and 0.842, respectively, Model 1 had a high positive predictive value; The negative predictive values of models 1, 2 and 3 were 0.840, 0.911, and 0.985, respectively, the negative predictive value of model 3 increased significantly; The accuracy of models 1, 2 and 3 were 0.859, 0.884, 0.940, Model 3 showed significantly improved accuracy, and the diagnostic efficacy of thyroid nodules was significantly improved (Fig. **7A** and **B**).

### 3.6 Nomogram of Model 3

Based on clinical ultrasound features (low/very low echo, irregular shape, microcalcification) and imageomics scores, a risk prediction nomogram for thyroid cancer was constructed (Fig. **8**). In the nomogram, internal microcalcification was the imaging feature with the highest predictive efficacy in this model. This nomogram demonstrated excellent discrimination and calibration, and could be effectively used for individualized risk assessment.

### 3.7 Validation of Model 3 Nomogram

The internal nomogram calibration analysis indicated that the difference between the calibration curve and the ideal curve was not significant, and suggested that Model 3 had good efficiency. Moreover, the nomogram showed good consistency, with an AUC of 0.978. The clinical decision curve results indicated that within the range of 0 - 0.85, Model 3 had a greater diagnostic advantage over the imageomics diagnostic model for patients with malignant thyroid nodules. The results showed that within the range of 0-0.85, Model 3 had more advantages over the imaging-based diagnostic model in diagnosing patients with malignant thyroid nodules (Fig. **9**).

## 4 DISCUSSION

In the field of medical diagnosis, ultrasound-based imageomics-based analysis has emerged as a cutting-edge technology [22, 23]. The analysis of ultrasound imaging biomarkers for thyroid nodules is a prominent example, demonstrating excellent diagnostic efficiency [24-26]. Despite these advancements, there were few studies on non-invasive multimodal ultrasound imaging combined with ultrasound elastography and supermicroscopic flow. This study developed and validated three diagnostic models for thyroid nodules: a clinical-ultrasound model (Model 1), a multimodal ultrasound imageomics model (Model 2), and a combined model integrating clinical features, ultrasound characteristics, and imageomics features (Model 3). While the combined model demonstrated excellent diagnostic performance (AUC 0.991 in training, 0.985 in validation), these results must be interpreted with caution, given the single-center retrospective design and the inherent challenges of imageomics research.

The results of this study showed that the AUC values of Model 1 in the training set and validation set were 0.914 and 0.907, respectively; the sensitivity was 0.875 and 0.877, respectively; and the specificity was 0.860 and 0.855, respectively. The overall diagnostic accuracy reached 86.7% (training set) and 86.7% (validation set). Among them, the LDA classifier performed outstandingly in terms of specificity (0.870) and accuracy (0.867). Although the AUC value of Model 1 was higher, its sensitivity was slightly lower than that of other models, indicating a potential risk of missed diagnosis. Previous studies had shown that a combined model based on clinical features and conventional ultrasound had good efficacy in the diagnosis of thyroid nodules [27]. Shi *et al.’s* study [28] demonstrated that a model combining age, gender, nodule size, and ultrasound boundary characteristics had an AUC of 0.89, which was consistent with the results of this study. However, the sensitivity of Model 1 in this study (0.877) was slightly lower than the 0.92 reported in the previous study, possibly due to the lower weight of microcalcification features included in this study. Additionally, this study found that nodular irregularity (*P*<0.001) and hypoechoic (*P*<0.001) were independent predictors of malignant nodules, which was consistent with the conclusion of Zhou *et al.’s* study [29], further verifying the core value of ultrasound features in the diagnosis of thyroid nodules. In conclusion, Model 1, by integrating clinical features and routine ultrasound examinations, could effectively diagnose thyroid nodules. The irregular shape and hypoechoic characteristics of the nodules were key indicators for determining their malignancy, which further highlights the diagnostic value of ultrasound examinations.

Model 2 was constructed based on multimodal ultrasound imaging biomarker features. The AUC values of the training set and validation set were 0.957 and 0.951 respectively; the sensitivity was 0.731 and 0.729, respectively; the specificity was 0.947 and 0.937 respectively; and the accuracy rate was 89.2% (training set) and 88.4% (validation set). The LDA classifier performs the best in terms of AUC (0.951), specificity (0.937), and accuracy rate (0.884). Although the sensitivity (0.729) was lower than that of Model 1, the specificity had significantly improved, indicating that Model 2 had a stronger ability to distinguish malignant nodules. Imaging biomarker technology has been widely applied in tumor diagnosis [30]. The study showed that the AUC of the thyroid nodule diagnosis model based on ultrasound imaging biomarkers could reach 0.94, which was consistent with the results of this study [31]. In this study, the AUC of Model 2 (0.951) was slightly higher than the 0.93 reported by them, possibly due to the inclusion of ultra-micro blood flow imaging (SMI) features in this study, which enhances the model’s ability to identify malignant nodules. In addition, this study found that 4183 imaging biomarker features had intra-group consistency (ICC>0.75), which was consistent with the conclusion of Li *et al.’s* study [32]. This indicated the stability of the feature extraction of the model. In conclusion, Model 2, which utilized multimodal ultrasound imaging biomarkers and SMI features, performs exceptionally well in tumor diagnosis, especially in the diagnosis of malignant nodules. This was attributed to its excellent AUC value, high specificity and accuracy, as well as stable feature extraction capabilities.

Model 3 integrates clinical features, conventional ultrasound features, and multimodal ultrasound imaging biomarker features. The AUC values of the training set and validation set were 0.991 and 0.985 respectively; the sensitivity was 0.961 and 0.957, respectively; the specificity was 0.949 and 0.934, respectively; and the accuracy rate was as high as 95.2% (training set) and 94.0% (validation set). The combined model significantly outperforms Model 1 and Model 2 in terms of sensitivity, specificity, negative predictive value (NPV), and accuracy rate (*P*<0.05). Model 3 demonstrated the best efficacy in both the training cohort and the validation cohort. The AUC values were 0.991 (95% confidence interval [CI]: 0.990-0.991) and 0.985 (95% CI: 0.974-0.996), respectively. The AUC values of Model 1 and Model 2 were lower than that of Model 3 (*P*<0.05), and indicated that the fusion of multimodal data could significantly improve diagnostic efficacy. Previous studies had shown that multimodal data fusion models had significant advantages in tumor diagnosis. Xu *et al.’s* [33] research demonstrated that the thyroid nodule diagnosis model combining clinical, ultrasound, and imageomics features achieved an AUC of 0.98, which was consistent with the results of this study. This might be due to the fact that more ultrasound features were included in this study, and a more rigorous feature selection process was adopted. Additionally, this study found that the NPV of the combined model (0.985) was significantly higher than that of Model 1 (0.840) and Model 2 (0.911), indicating its higher reliability in excluding benign nodules.

This study employed a traditional imageomics workflow: hand-crafted feature extraction followed by machine learning classification. The primary advantage of this approach is interpretability—clinicians can understand which specific features (*e.g*., texture entropy, gray-level mean) drive predictions, and coefficients can be examined for biological plausibility. The LDA classifier used in Model 3 provides a transparent linear combination of features, and we have provided the full model formula in the Appendix to enable external validation. However, traditional imageomics has inherent limitations. Feature extraction relies on predefined mathematical formulas that may not capture all diagnostically relevant information. Deep learning approaches, particularly convolutional neural networks (CNNs), can learn hierarchical features directly from images without manual segmentation or feature engineering [34]. Recent studies suggest that deep learning models often outperform traditional imageomics in medical image analysis, especially with large datasets.

The results of this study showed that when the constructed nomogram was used for the diagnosis of thyroid nodules, the corresponding index value presented by the internal calibration analysis of the nomogram reached 0.978, and the calibration curve did not differ much from the ideal curve. This indicated that the nomogram was highly consistent with the combined diagnostic results, and Model 3 had good diagnostic efficacy. However, different models vary in the treatment benefits reflected by the clinical decision curve. In the clinical decision curve analysis, for all diagnostic models, the benefits for patients with malignant thyroid nodules after treatment were greater than those without treatment within each threshold range. Within the range of 0 to 0.85, Model 3 had a greater advantage in diagnosing patients with malignant thyroid nodules compared to Diagnostic Model 1 and Model 2, which were based on imaging and clinical features. Zhao [35] *et al*. studied that the efficacy of different diagnostic models in specific disease diagnosis varies due to the different model construction methods and included features, which was partially consistent with the results of this study. In this study, the nomogram was highly consistent with the combined diagnostic results, which was due to the fact that during the construction of the nomogram, not only the clinical routine ultrasound features (such as hypoechoic or extremely hypoechoic, irregular shape, and microcalcification) selected from the combined diagnostic model results were included, but also the imageomics scores were combined. This comprehensive feature inclusion method enabled the nomogram to more comprehensively and accurately reflect the pathological features of thyroid nodules, thereby improving the diagnostic consistency [36]. However, different models had differences in the treatment benefits reflected by the clinical decision curve, possibly because the features and algorithms of each model were different [37]. Model 3 combined multiple features to construct the nodal graph. Within the 0-0.85 threshold range, it could fully demonstrate the advantages of its comprehensive features, resulting in a diagnostic effect that was superior to Model 1 and Model 2. This was of great significance for improving the diagnostic accuracy of thyroid nodules and the treatment outcome of patients.

A fundamental barrier to clinical translation of imageomics models is feature reproducibility [30]. Imageomics features are sensitive to multiple factors, including segmentation variability, image acquisition parameters, scanner differences, and preprocessing techniques [32]. In this study, we addressed this challenge through several measures: (1) two experienced sonographers independently segmented all nodules with blinding to clinical data; (2) only features with inter- and intra-observer ICC>0.75 were retained; (3) Dice similarity coefficients (mean 0.89±0.05) confirmed good segmentation agreement. Despite these precautions, several reproducibility concerns remain. First, manual segmentation, even with high ICC, is inherently subjective and time-consuming, limiting clinical scalability. Second, all images were acquired using a single ultrasound machine (Philips EPIQ 7) with standardized settings; feature stability across different equipment and protocols remains unknown. Third, we only assessed reproducibility within a single center; multi-center, multi-vendor studies are essential to identify truly robust imageomics biomarkers. Future research should incorporate test-retest analysis and explore harmonization techniques such as ComBat to reduce batch effects.

## 5 STUDY LIMITATIONS

Several limitations of this study should be addressed. First, the single-center retrospective design and limited sample size restrict generalizability. Future work must include multi-center prospective cohorts to validate the model's external validity; Second, image selection bias—operators tended to choose the “best” image—may overestimate performance. To mitigate this, future studies should use systematic sampling or analyze all available images per nodule. Third, manual segmentation introduces observer variability and is time-consuming. Although inter-observer agreement was acceptable (ICC>0.75), intra-observer variability was not assessed. We plan to develop automated segmentation algorithms to enhance reproducibility and clinical workflow integration. Fourth, the high dimensionality of imageomics features relative to sample size raises the risk of overfitting, despite rigorous feature selection. Independent external validation is essential to confirm true model performance. Fifth, we did not compare our model with the widely used TIRADS system. Future studies should perform head-to-head comparisons to demonstrate incremental clinical value; Sixth, cost-effectiveness was not evaluated. A formal health-economic analysis will be conducted to assess potential reductions in unnecessary biopsies and healthcare costs. Finally, we only explored hand-crafted imageomics features; deep learning approaches may capture complementary information. Subsequent research will investigate hybrid imageomics-deep learning models for improved accuracy and automation. Addressing these limitations will move this model closer to clinical implementation.

## CONCLUSION

The diagnostic model based on multimodal ultrasound imaging and clinical features could significantly improve the diagnostic efficacy of thyroid nodules, reduce unnecessary biopsy or surgery, and have certain clinical application value. The combined diagnostic model nomogram allows a more intuitive assessment of the malignant risk of thyroid nodules. The application of the clinical decision curve could obtain the best net benefit and has good clinical application value.

## Figures and Tables

**Fig. (1):**
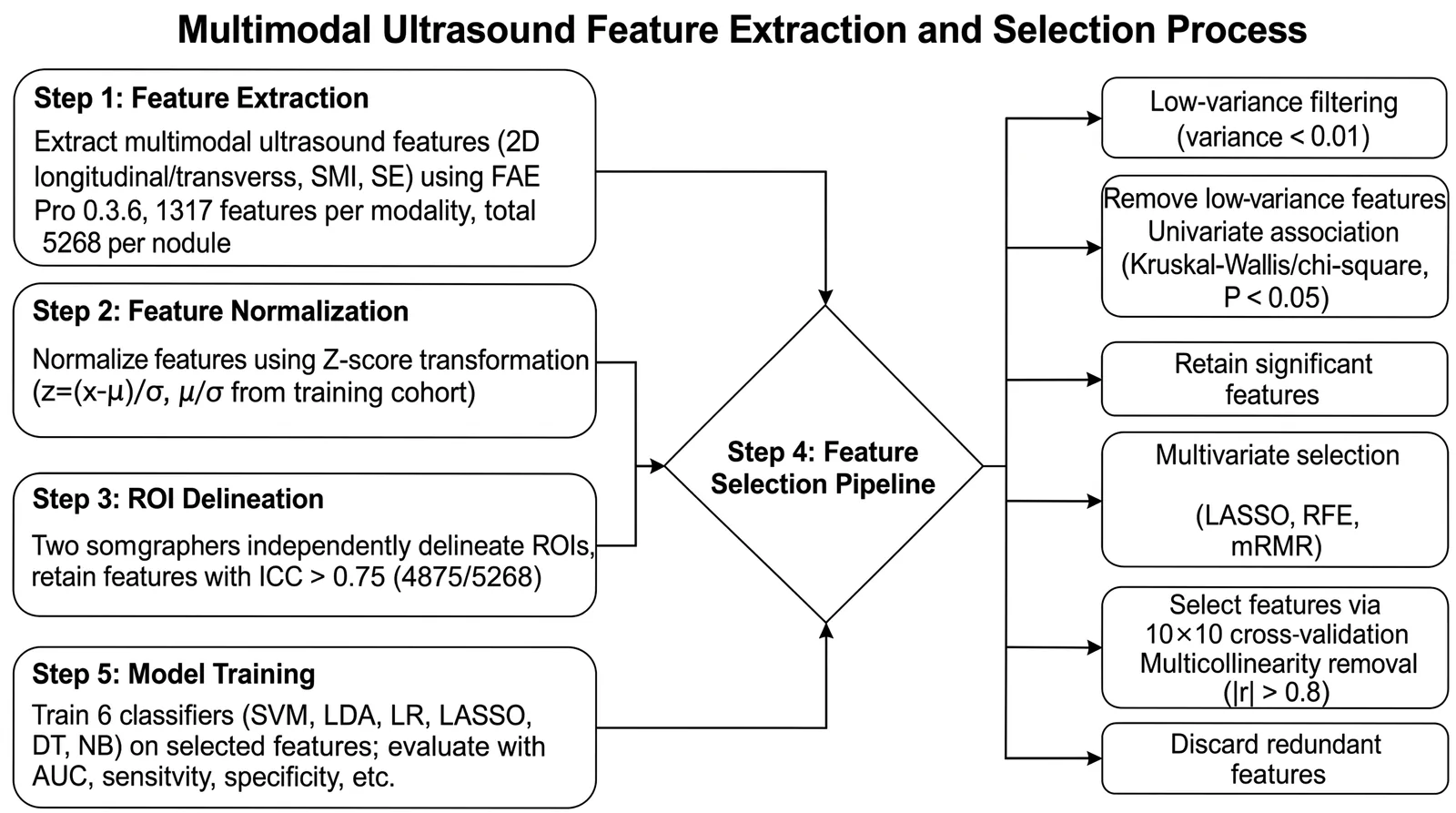
Feature extraction flowchart.

**Fig. (2):**
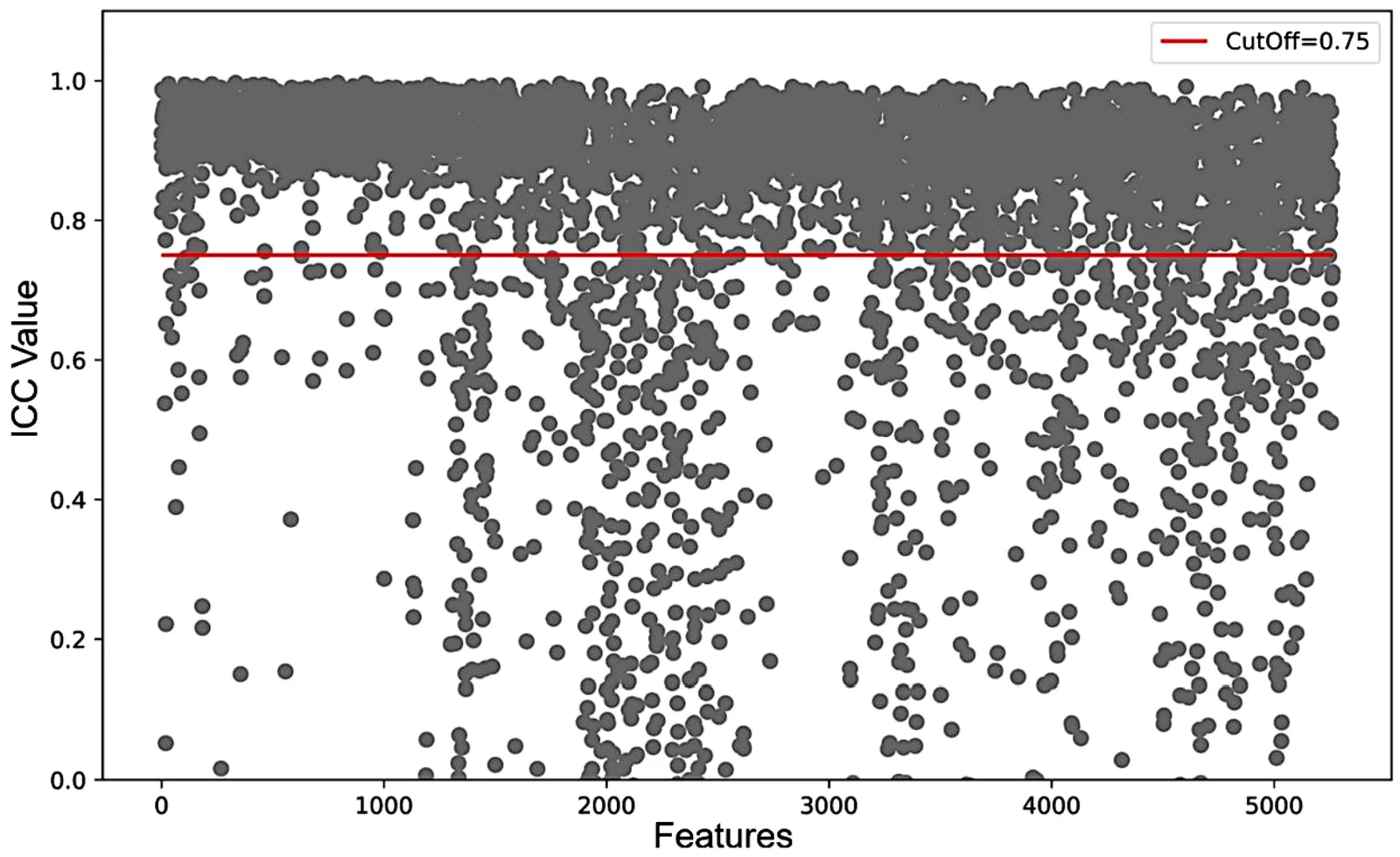
Intraclass correlation coefficients (ICCs) for Physicians **A** and **B**.

**Fig. (3):**
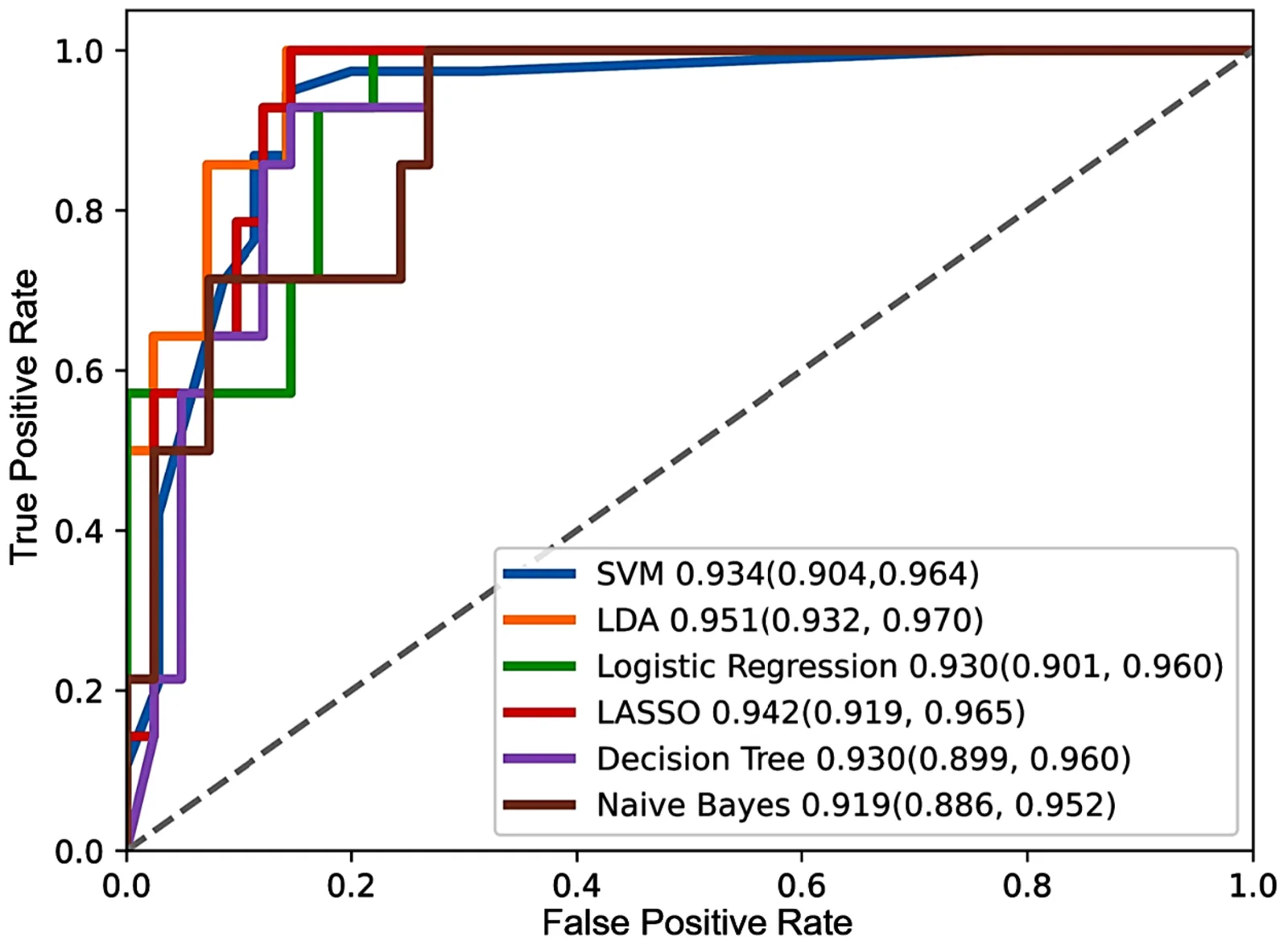
Receiver-operating characteristic curves of Model 2 for the six classifiers.

**Fig. (4):**
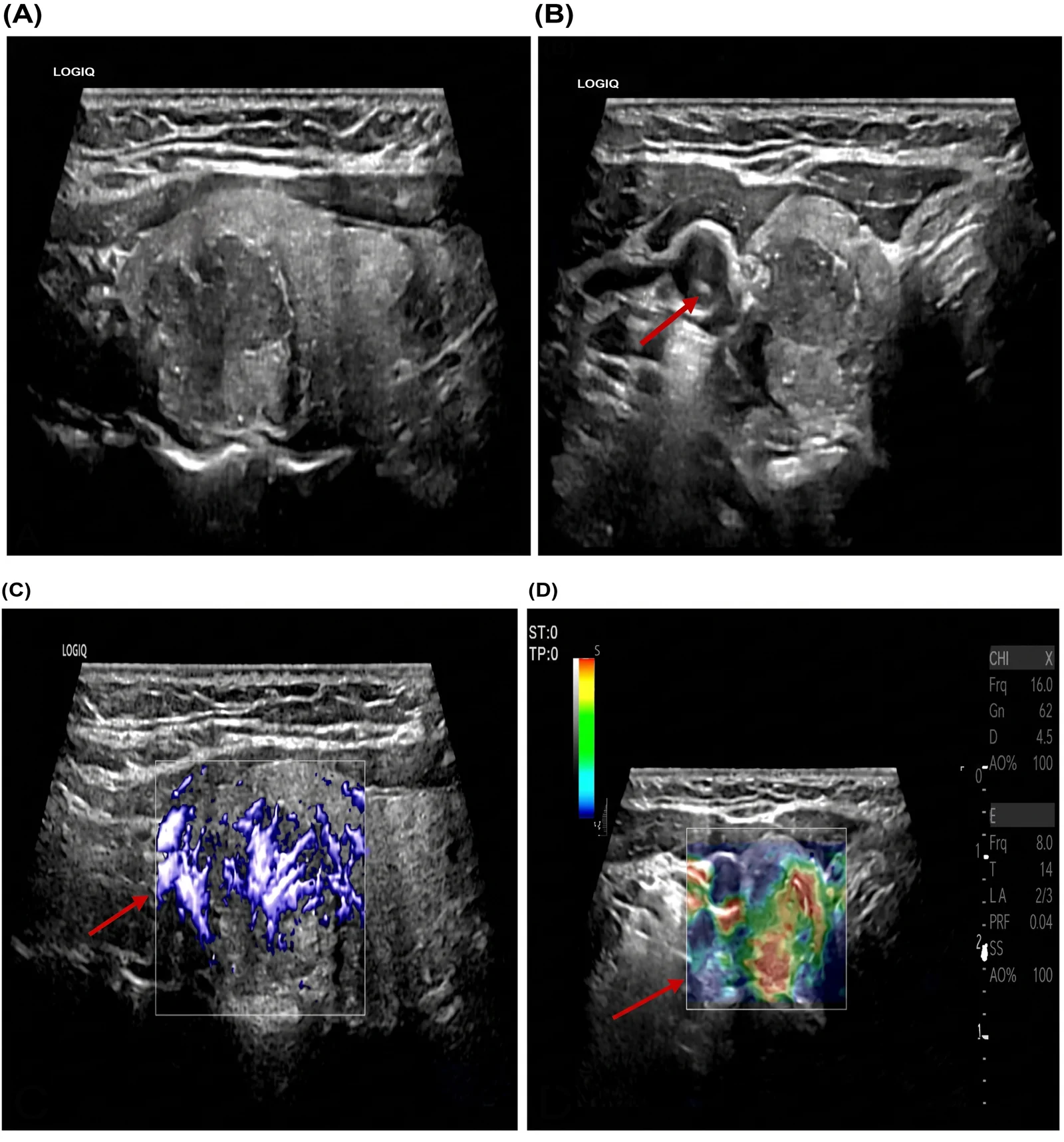
Multimodal ultrasound images. (**A**) Longitudinal image of thyroid nodule; (**B**) Transverse image of thyroid nodule; (**C**) SMI image of thyroid nodule; (**D**) SE image of thyroid nodule.

**Fig. (5):**
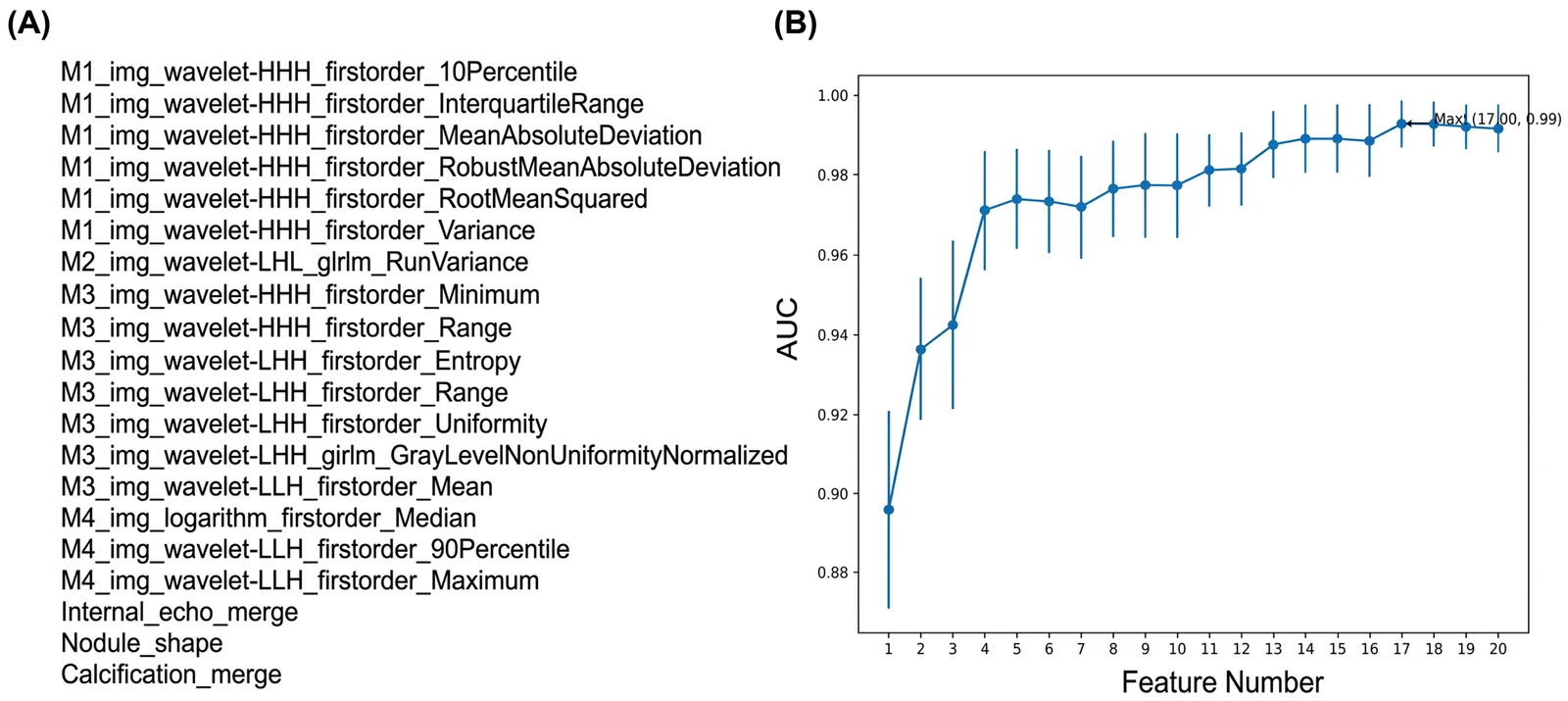
(**A**) Summary table of typical characteristic names of Model 3. (**B**) Summary table of the cross-validated areas under the curve.

**Fig. (6):**
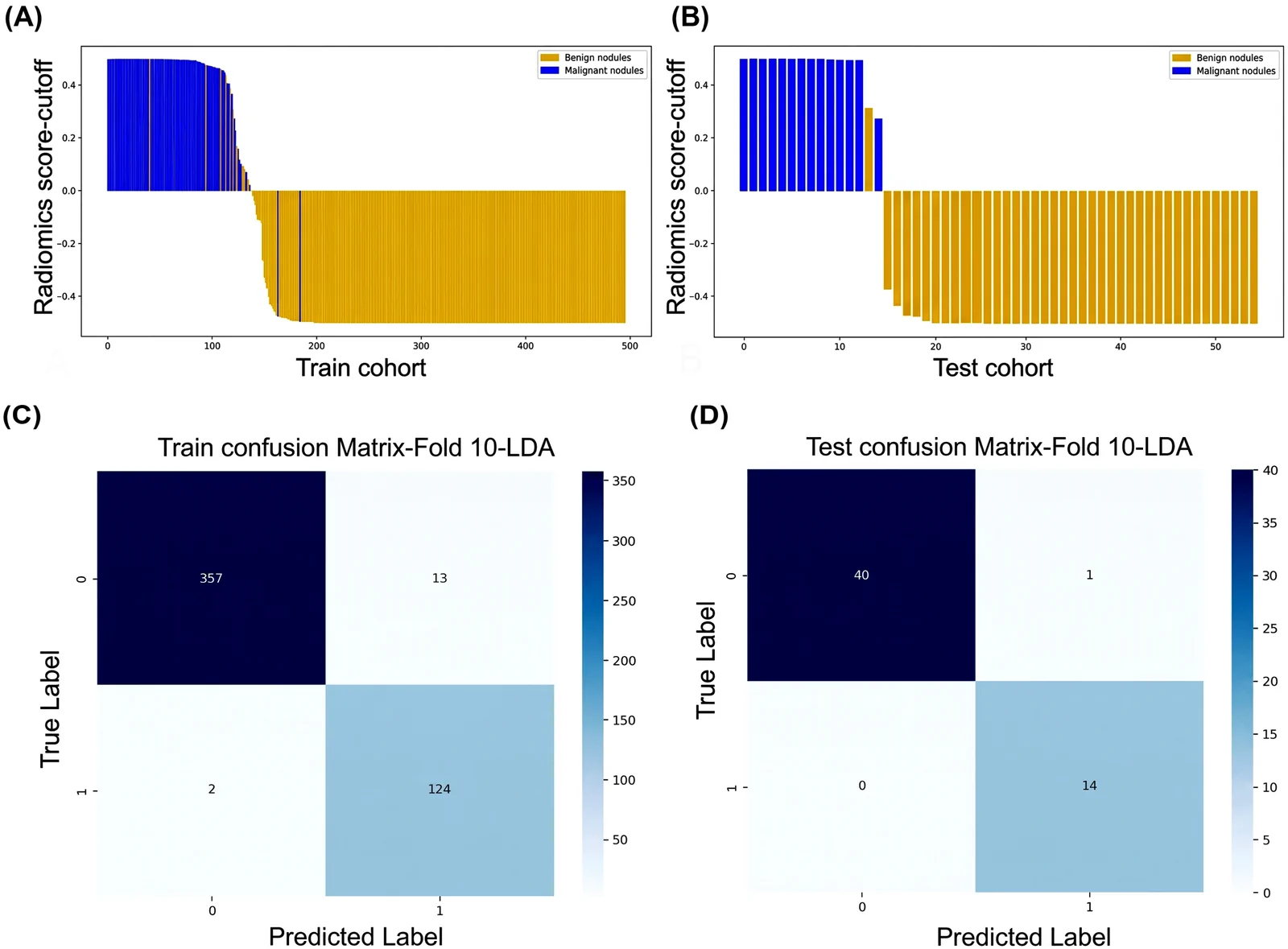
Confusion matrix of Model 3 and its verification. (**A**, **B**) represent the best display effects of the confusion matrix of Model 3. The x-axis represents the number of patients, and the y-axis represents the diagnosis results and their corresponding differences. If the difference is positive, the lesion is identified as malignant; if the difference is negative, the lesion is identified as benign. In the above figure, blue and orange respectively represent malignant and benign lesions. (**C**, **D**) are the classification and error counts obtained after diagnosis using Model 3 on the training set and validation set. The predicted labels are diagnostic indicators, while the true labels are pathological indicators.

**Fig. (7):**
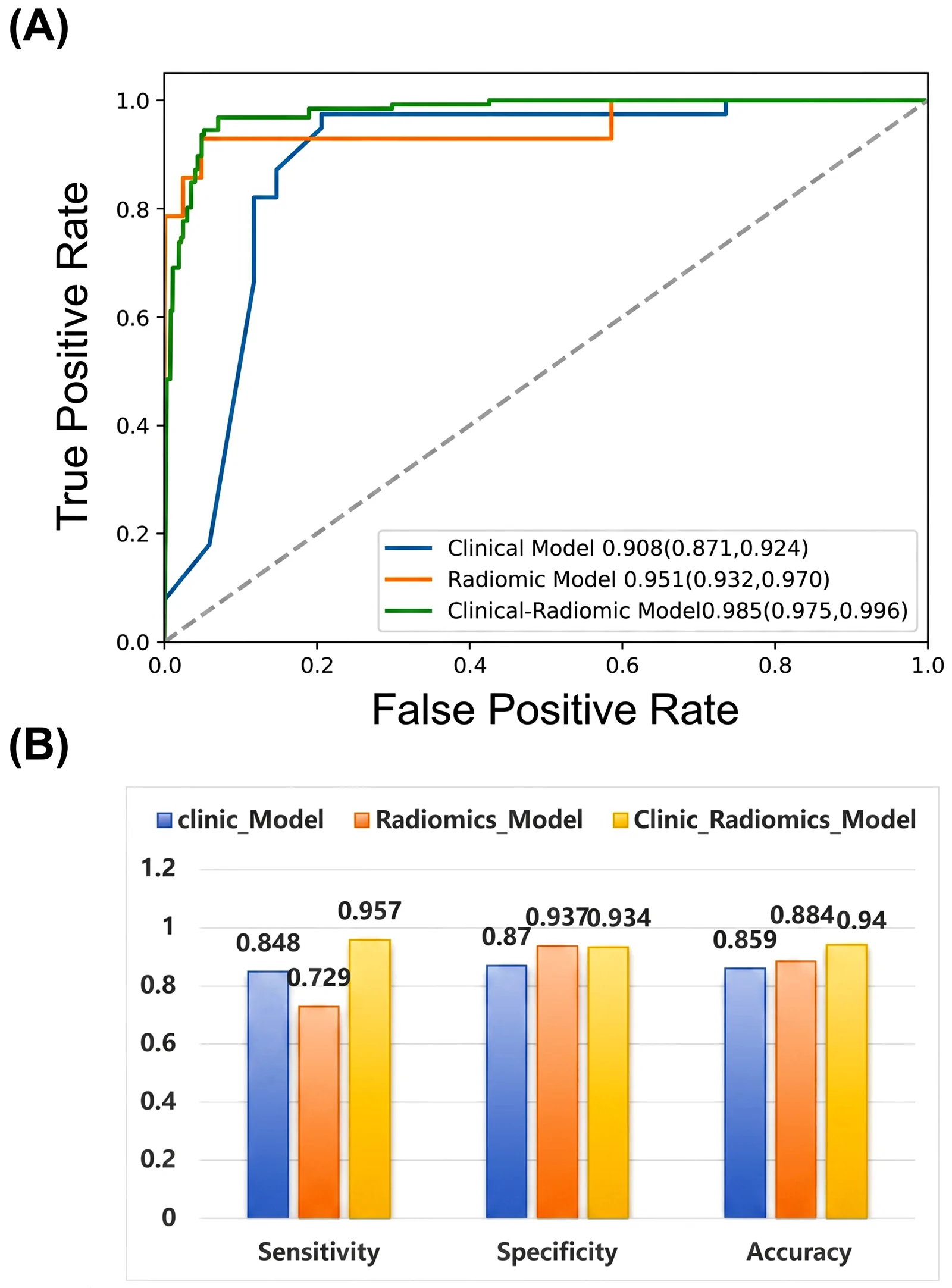
The ROC curves of the three models (**A**) and the differences among these three models (**B**).

**Fig. (8):**
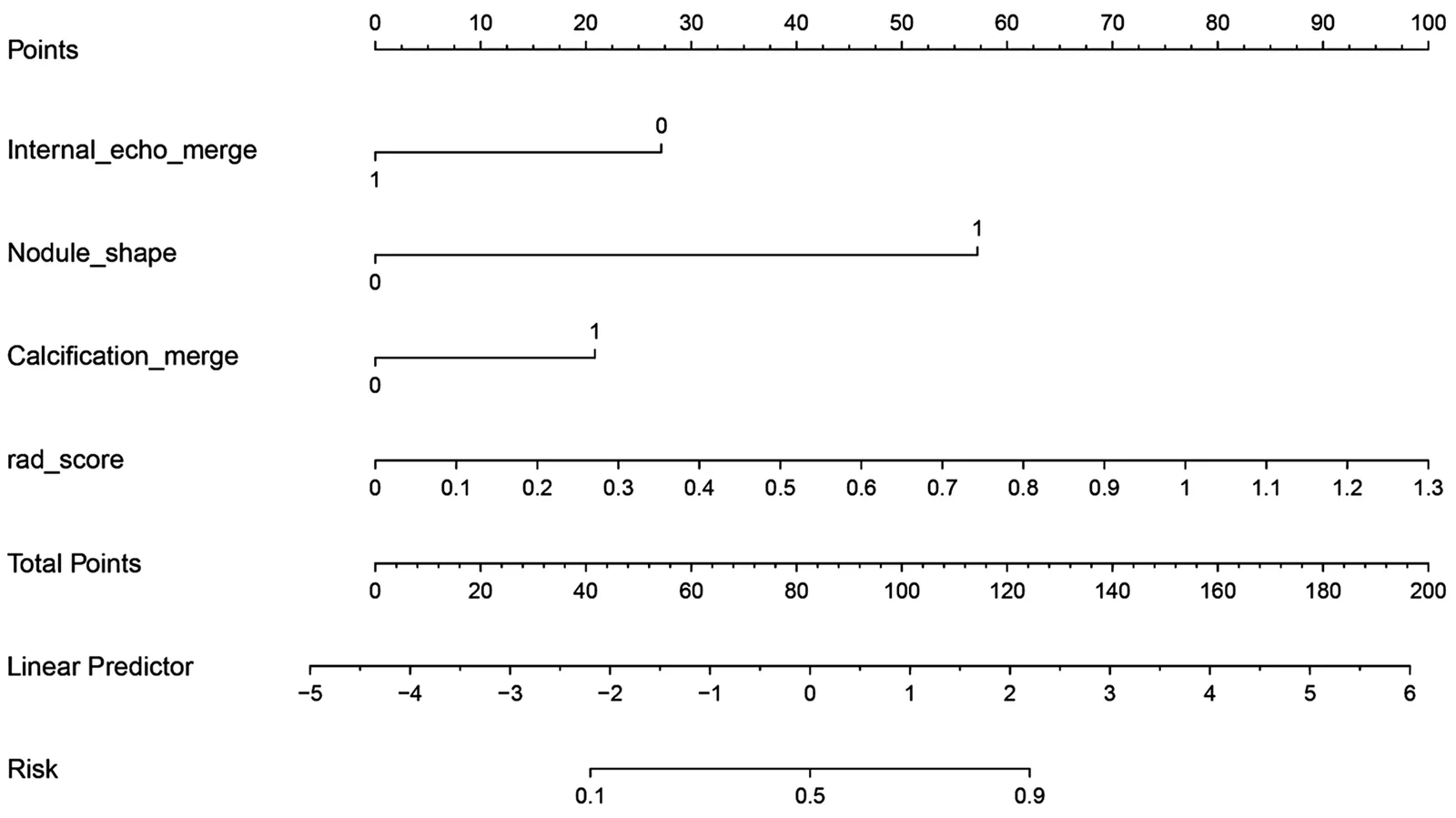
Model 3 nomogram-validation cohort.

**Fig. (9):**
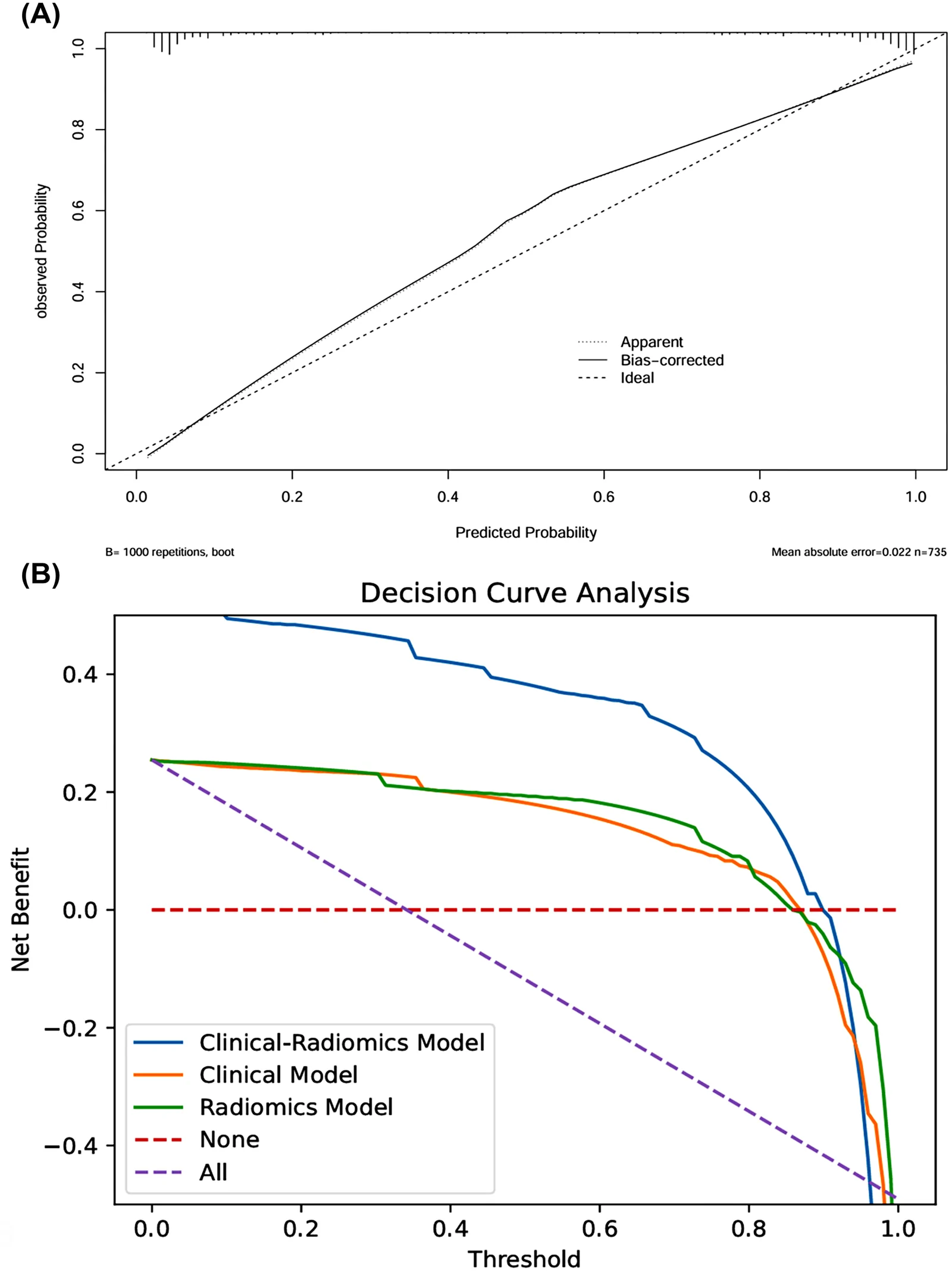
(**A**) bar chart calibration curve, bias-corrected and ideal results were reported for the diagnostic curve, calibration diagnostic curve, and standard curve. (**B**) clinical decision curve of the diagnostic model, the x-axis represents the risk threshold, and the y-axis represents the net benefit.

**Table 1:** Comparison of baseline data in the training set and the validation set.

**Cohort Item**	**Training Cohort (n=514)**		***P***	**Validation Cohort (n=221)**		***P***
	**Malignancy (n=272)**	**Benign (n=242)**		**Malignancy (n=117)**	**Benign (n=104)**	
Sex	-	-	0.886	-	-	0.106
Male	61 (0.22)	53 (0.22)		18 (0.15)	25 (0.24)	
Female	211 (0.78)	189 (0.78)		99 (0.85)	79 (0.76)	
BMI (kg/m^2^)	23.60±2.99	23.21±2.93	0.141	23.41±2.71	23.43±2.79	0.260
Smoking history	100 (0.37)	80 (0.29)	0.379	42 (0.36)	36 (0.34)	0.842
Allergy history	170 (0.62)	140 (0.58)	0.282	69 (0.59)	62 (0.60)	0.923
Boundary	-	-	0.922	-	-	0.891
Clear	200 (0.74)	211 (0.87)	-	71 (0.61)	92 (0.88)	-
Unclear	72 (0.26)	31 (0.13)	-	46 (0.39)	12 (0.12)	-

**Table 2:** Clinical manifestations and basic ultrasound characteristics of patients with nodules.

**Cohort Item**	**Training Cohort (n=514)**		***P***	**Validation Cohort (n=221)**		***P***
	**Malignancy (n=272)**	**Benign (n=242)**		**Malignancy (n=117)**	**Benign (n=104)**	
Age ( 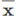 ±s)	47.12±10.99	52.53±11.58	<0.001	46.72±10.09	54.01±12.57	<0.001
≤55	213 (0.78)	137 (0.57)	-	87 (0.74)	52 (0.5)	-
>55	59 (0.22)	105 (0.43)	-	30 (0.26)	52 (0.5)	-
Echo	-	-	<0.001	1	-	<0.001
Low/very low	252 (0.93)	185 (0.76)	-	107 (0.91)	87 (0.84)	-
Equal/high/mixed	20 (0.07)	57 (0.24)	-	10 (0.09)	17 (0.16)	-
Boundary	-	-	<0.001	2	9	<0.001
Sharp	46 (0.17)	220 (0.91)	-	20 (0.17)	96 (0.92)	-
Unsharp	226 (0.83)	20 (0.09)	-	97 (0.83)	8 (0.08)	-
Shape	-	-	<0.001	-	-	<0.001
Rule	33 (0.12)	220 (0.91)	-	15 (0.13)	96 (0.92)	-
Unrule	239 (0.88)	20 (0.09)	-	102 (0.87)	8 (0.08)	-
Aspect ratio(A/T)	-	-	<0.001	8	8	<0.001
>1	170 (0.62)	14 (0.06)	-	80 (0.68)	8 (0.08)	-
≤1	102 (0.37)	228 (0.94)	-	37 (0.32)	96 (0.92)	-
Microcalcification	-	-	<0.001	-	-	<0.001
Yes	776 (0.28)	4 (0.02)	-	28 (0.24)	7 (0.07)	-
No	196 (0.72)	238 (0.98)	-	89 (0.76)	97 (0.93)	-
Mulifocaliy	-	-	<0.001	-	-	0.033
Yes	171 (0.63)	193 (0.8)	-	72 (0.62)	78 (0.75)	-
No	101 (0.37)	49 (0.2)	-	45 (0.38)	26 (0.25)	-
Super microflow	-	-	0.005	-	-	0.874
I+II	156 (0.57)	109 (0.45)	-	64 (0.55)	58 (0.56)	-
III+IV	116 (0.43)	133 (0.55)	-	53 (0.45)	46 (0.44)	-

**Table 3:** Multivariate logistic regression analysis of all nodules.

**Items**	**β**	**S.E**	**Wald**	***P***	**OR**	**OR 2.5%**	**OR 97.5%**
Age_group	-0.864	0.340	6.448	0.011	0.422	0.216	0.821
Nodule_size_group2	-0.562	0.352	2.56	0.110	0.570	0.286	1.135
Internal_echo_merge	-1.828	0.369	24.529	<0.001	0.161	0.078	0.331
Boundary	-0.583	0.864	0.456	0.500	0.558	0.103	3.034
Nodule_shape	3.157	0.839	14.151	0.000	23.509	4.537	121.813
Aspect_ratio	0.536	0.4502	1.418	0.234	1.709	0.707	4.131
Calcification_merge	1.221	0.616	3.925	0.048	3.390	1.013	11.345
Nodule_number_group	-0.755	0.345	4.780	0.029	0.470	0.239	0.925
SMI_merge	-0.077	0.315	0.059	0.807	0.926	0.500	1.717
Const	0.266	0.446	0.354	0.552	1.304	0.544	3.127

**Table 4:** Performance evaluation of the six classifiers in the clinical models.

**Mode-** **clinical**	**SVM**	**LDA**	**LR**	**LASSO**	**DT**	**NB**
VIF	1.414					
AUC	-	-	-	-	-	-
Train	0.889	0.913	0.914	0.914	0.928	0.910
Test	0.858	0.908	0.907	0.907	0.891	0.908
Sensitivity	-	-	-	-	-	-
Train	0.958	0.853	0.875	0.875	0.931	0.832
Test	0.936	0.848	0.877	0.877	0.892	0.830
Specificity	-	-	-	-	-	-
Train	0.787	0.869	0.860	0.860	0.821	0.870
Test	0.771	0.870	0.855	0.855	0.798	0.867
PPV	-	-	-	-	-	-
Train	0.836	0.880	0.875	0.875	0.855	0.878
Test	0.822	0.880	0.872	0.872	0.834	0.876
NPV	-	-	-	-	-	-
Train	0.946	0.840	0.859	0.859	0.916	0.822
Test	0.920	0.840	0.864	0.864	0.872	0.826
Accuracy	-	-	-	-	-	-
Train	0.878	0.860	0.868	0.868	0.879	0.850
Test	0.859	0.859	0.867	0.867	0.848	0.848

**Abbreviations:** VIF, Variance Inflation Factor; AUC, area under the curve; PPV, positive predictive value; NPV, negative predictive value; LR, logistic regression; SVM, support vector machine; NB, naive Bayes; DT, decision tree; LASSO, least absolute shrinkage and selection operator; LDA, and linear discriminant analysis.

**Table 5:** Diagnostic efficacy of the six classifiers used in multimodal imaging omics.

** Mode- ** ** imageomics **	**SVM**	**LDA**	**LR**	**LASSO**	**DT**	**NB**
VIF	1.710					
AUC	-	-	-	-	-	-
Train	0.950	0.957	0.936	0.946	0.964	0.921
Test	0.934	0.951	0.930	0.942	0.930	0.919
Sensitivity	-	-	-	-	-	-
Train	0.680	0.731	0.633	0.662	0.937	0.771
Test	0.621	0.729	0.629	0.657	0.914	0.764
Specificity	-	-	-	-	-	-
Train	0.962	0.947	0.932	0.942	0.927	0.878
Test	0.949	0.937	0.920	0.922	0.927	0.876
PPV	-	-	-	-	-	-
Train	0.860	0.825	0.760	0.797	0.813	0.683
Test	0.828	0.814	0.761	0.785	0.822	0.704
NPV	-	-	-	-	-	-
Train	0.899	0.912	0.882	0.891	0.977	0.918
Test	0.880	0.911	0.879	0.888	0.971	0.917
Accuracy	-	-	-	-	-	-
Train	0.891	0.892	0.856	0.871	0.929	0.851
Test	0.866	0.884	0.846	0.855	0.924	0.848

**Abbreviations:** VIF, Variance Inflation Factor; AUC, area under the curve; PPV, positive predictive value; NPV, negative predictive value; LR, logistic regression; SVM, support vector machine; NB, naive Bayes; DT, decision tree; LASSO, least absolute shrinkage and selection operator; LDA, and linear discriminant analysis.

**Table 6:** Summary of the efficacy evaluation results of the diagnostic models.

**Model**	** Clinical Model **	** Imageomics Model **	** Clinical-Imageomics Model **
AUC	-	-	-
Train	0.913	0.957	0.991
Test	0.908	0.951	0.985
Sensitivity	-	-	-
Train	0.853	0.731	0.961
Test	0.848	0.729	0.957
Specificity	-	-	-
Train	0.869	0.947	0.949
Test	0.870	0.937	0.934
PPV	-	-	-
Train	0.880	0.825	0.866
Test	0.880	0.814	0.842
NPV	-	-	-
Train	0.840	0.912	0.986
Test	0.840	0.911	0.985
Accuracy	-	-	-
Train	0.860	0.892	0.952
Test	0.859	0.884	0.940

## Data Availability

In this study, the generated and/or analyzed data, due to reasons related to patient privacy protection and institutional policies, are not yet made available to the public. However, the relevant researchers can obtain the data from the corresponding author [Z.Z] upon reasonable request.

## LIST OF ABBREVIATIONS

PTC: Papillary Thyroid Carcinoma
ACR: American College of Radiology
TIRADS: Thyroid Imaging Reporting and Data System
SE: Strain Elastography
SWE: Shear Wave Elastography
SMI: Superb Microvascular Imaging
ROI: Region of Interest
ICC: Intraclass Correlation Coefficient
LR: Logistic Regression
SVM: Support Vector Machine
NB: Naive Bayes
DT: Decision Tree
LASSO: Least Absolute Shrinkage and Selection Operator
LDA: Linear Discriminant Analysis
AUC: Area Under the Curve
ROC: Receiver Operating Characteristic Curve

## STANDARDS OF REPORTING

This study adheres to the “Transparent Reporting of Individualized Predictive Models for Diagnosis or Prognosis” statement (TRIPOD).

## Appendix

**Appendix Table A1:** Features included in the combined diagnostic model.

**Feature**	**Definition**	**Coefficient (β)**
SMI texture entropy	Entropy of texture features on Superb Microvascular Imaging (SMI)	0.821
2D longitudinal gray mean	Mean gray-scale value in the longitudinal plane of 2D ultrasound	0.763
SE skewness	Skewness of strain elastography (SE) image	0.708
Microcalcification	Presence of microcalcifications (0=no, 1=yes)	0.612
Age≥55 years	Age 55 years or older (0=no, 1=yes)	0.587
Shape irregular	Irregular shape on B-mode ultrasound (0=regular, 1=irregular)	0.452
Margin non-defined	Non-defined or infiltrative margin (0=defined, 1=non-defined)	0.498
Taller-than-wide	Anteroposterior dimension > transverse dimension (0=no, 1=yes)	0.523
Composition solid	Solid composition (0=cystic/spongiform, 1=solid)	0.376
Echogenicity hypo	Hypoechoic or markedly hypoechoic (0=iso/hyper, 1=hypo)	0.441
SMI vascular grade	SMI vascularity grade (0=absence, 1=peripheral, 2=penetrating)	0.509
2D transverse skewness	Skewness of gray distribution in transverse 2D plane	0.394
SE mean strain	Mean strain value on elastography	0.467
Imageomics GLCM contrast	Contrast from grey-level co-occurrence matrix (GLCM)	0.521
Imageomics GLRLM RUN	Run length non-uniformity from GLRLM	0.482
Imageomics shape flatness	Flatness of nodule shape	0.415
Imageomics ngtdm busyness	Busyness from neighbourhood grey-tone difference matrix	0.438
TSH level	Serum TSH level (µIU/mL)	0.299
Family history	Family history of thyroid cancer (0=no, 1=yes)	0.355
Nodule size mm	Maximum diameter of nodule (mm)	0.371

**
Appendix: Full model formula for the combined diagnostic model
**

1. Linear predictor (LP)

The linear predictor is calculated as:

LP=β0+β1X1+β2X2+···+β20X20LP=β0 +β1 X1 +β2 X2 +···+β20 X20

where:

• X1X1 to X20X20 are the selected features (normalized using Z-scores derived from the training set);

• β0β0 is the intercept;

• β1β1 to β20β20 are the regression coefficients.

2. Predicted probability of malignancy

P=11+e−LPP=1+e−LP1

A probability P≥0.5 classifies the thyroid nodule as malignant (cut-off may be adjusted according to clinical need).

3. Feature list and coefficients

All continuous features were standardized using the training set mean and standard deviation: Xstandardized=(Xraw−μtrain)/σtrainXstandardized =(Xraw −μtrain )/σtrain .

## References

[1] Roman B.R., Morris L.G., Davies L. (2017). The thyroid cancer epidemic, 2017 perspective.. Curr. Opin. Endocrinol. Diabetes Obes..

[2] Abdullah M.I., Junit S.M., Ng K.L., Jayapalan J.J., Karikalan B., Hashim O.H. (2019). Papillary thyroid cancer: Genetic alterations and molecular biomarker investigations.. Int. J. Med. Sci..

[3] Ruan X., Yang F., Li M., Dong Q., Hao W., Zhang W., Yun X., Li D., Zhao J., Qin X., Rong Z., Guo T., Wang L., Pan Y., Gao M., Zhang M., Zheng X. (2025). Multi-Omics-Based characterization of DNA methylation episignatures for papillary thyroid cancer in a chinese population.. Thyroid.

[4] Liu F., Wang Y., Xiong Y., Li X., yao J., Ju H., Ren F., Zhang L., Wang H. (2024). Diagnostic value of combined ultrasound contrast and elastography for differentiating benign and malignant thyroid nodules: A meta-analysis.. Sci. Rep..

[5] Hu Y., Xu S., Zhan W. (2022). Diagnostic performance of C-TIRADS in malignancy risk stratification of thyroid nodules: A systematic review and meta-analysis.. Front. Endocrinol..

[6] Omakobia E., McClean S., England J., Walden A. (2021). Is sonographic intra-nodular vascularity a reliable predictor of thyroid malignancy? A UK tertiary centre study.. J. Laryngol. Otol..

[7] Qi Q., Zhou A., Guo S., Huang X., Chen S., Li Y., Xu P. (2021). Explore the diagnostic efficiency of Chinese thyroid imaging reporting and data systems by comparing with the other four systems (ACR TI-RADS, Kwak-TIRADS, KSThR-TIRADS, and EU-TIRADS): A single-center study.. Front. Endocrinol..

[8] Li H., Bhatt M., Qu Z., Zhang S., Hartel M.C., Khademhosseini A., Cloutier G. (2022). Deep learning in ultrasound elastography imaging: A review.. Med. Phys..

[9] Mehanna H., Deeks J.J., Boelaert K., Madani G., Sidhu P., Nankivell P., Sharma N., Woolley R., Taylor J., Fulton-Lieuw T., Palmer A. (2024). Real-time ultrasound elastography in the diagnosis of newly identified thyroid nodules in adults: The ElaTION RCT.. Health Technol. Assess..

[10] Cao Y., Zhong X., Diao W., Mu J., Cheng Y., Jia Z. (2021). Radiomics in differentiated thyroid cancer and nodules: Explorations, application, and limitations.. Cancers.

[11] Gitto S., Messina C., Chianca V., Tuscano B., Lazzara A., Corazza A., Pedone L., Albano D., Sconfienza L.M. (2020). Superb microvascular imaging (SMI) in the evaluation of musculoskeletal disorders: A systematic review.. Radiol. Med..

[12] Negroni D., Maddalena G., Bono R., Abruzzese F., Cesano S., Conte P., Airoldi C., Neri P., Carriero A. (2024). Superb microvascular imaging (SMI) and elastosonography in thyroid nodule: Diagnostic value in a real-time cohort.. J. Ultrasound.

[13] Wang J., Liu J., Liu Z. (2019). Impact of ultrasound-guided fine needle aspiration cytology for diagnosis of thyroid nodules.. Medicine.

[14] He T., Fong J.N., Moore L.W., Ezeana C.F., Victor D., Divatia M., Vasquez M., Ghobrial R.M., Wong S.T.C. (2021). An imageomics and multi-network based deep learning model for risk assessment of liver transplantation for hepatocellular cancer.. Comput. Med. Imaging Graph..

[15] Kwon M., Shin J.H., Park H., Cho H., Kim E., Hahn S.Y. (2020). Radiomics based on thyroid ultrasound can predict distant metastasis of follicular thyroid carcinoma.. J. Clin. Med..

[16] Yoon J.H., Han K., Lee E., Lee J., Kim E.K., Moon H.J., Park V.Y., Nam K.H., Kwak J.Y. (2020). Radiomics in predicting mutation status for thyroid cancer: A preliminary study using radiomics features for predicting BRAFV600E mutations in papillary thyroid carcinoma.. PLoS One.

[17] Abbasian Ardakani A., Gharbali A., Mohammadi A. (2015). Application of texture analysis method for classification of benign and malignant thyroid nodules in ultrasound images.. Iran. J. Cancer Prev..

[18] Abbasian Ardakani A., Mohammadi A., Yeong C.H., Ng W.L., Ng A.H., Tangaraju K.N., Behestani S., Mirza-Aghazadeh-Attari M., Suresh R., Acharya U.R. (2025). Diagnosis of thyroid nodule malignancy using peritumoral region and artificial intelligence.. J. Ultrasound Med..

[19] Liang Y., Huang X., Song Z., Yang Y., Lei J., Ren M., Tan L., Zhang H. (2022). Clinical study of ultrasonic evaluation of T/N staging of differentiated thyroid carcinoma using AJCC 8th staging criteria.. PLoS One.

[20] Zhao H., Tang X.Z., Wang D.P., Yu P.C., He C., Chen X.F., Ye W.D., Wu J.H., Hu J.Q., Wang Y.L. (2025). Prognostic factors and survival outcomes in thyroid cancer patients with bone metastasis: Insights from a Population-Based cohort study.. Endocr. Pract..

[21] Zhou P., Chen F., Zhou P., Xu L., Wang L., Wang Z., Yu Y., Liu X., Wang B., Yan W., Zhou H., Tao Y., Liu W. (2023). The use of modified TI-RADS using contrast-enhanced ultrasound features for classification purposes in the differential diagnosis of benign and malignant thyroid nodules: A prospective and multi-center study.. Front. Endocrinol..

[22] Yan D., Li Q., Lin C.W., Shieh J.Y., Weng W.C., Tsui P.H. (2024). Hybrid QUS radiomics: A multimodal-integrated quantitative ultrasound radiomics for assessing ambulatory function in duchenne muscular dystrophy.. IEEE J. Biomed. Health Inform..

[23] Bandara M.S., Gurunayaka B., Lakraj G., Pallewatte A., Siribaddana S., Wansapura J. (2022). Ultrasound based radiomics features of chronic kidney disease.. Acad. Radiol..

[24] Zhang D., Yang F., Hou W., Wang Y., Mu J., Wang H., Wei X. (2025). Ultrasonic radiomics in predicting pathologic type for thyroid cancer: A preliminary study using radiomics features for predicting medullary thyroid carcinoma.. Front. Endocrinol..

[25] Saini M., Larson N.B., Fatemi M., Alizad A. (2025). Deep learning based motion correction in ultrasound microvessel imaging approach improves thyroid nodule classification.. Sci. Rep..

[26] Macvanin M.T., Gluvic Z.M., Zaric B.L., Essack M., Gao X., Isenovic E.R. (2023). New biomarkers: Prospect for diagnosis and monitoring of thyroid disease.. Front. Endocrinol..

[27] Xu C., Fang J., Li W., Sun C., Li Y., Lowe S., Bentley R., Chen S., He C., Li X., Wang B., Yin C., Li W., Li W. (2022). Construction and validation of BRAF mutation diagnostic model based on ultrasound examination and clinical features of patients with thyroid nodules.. Front. Genet..

[28] Shi X., Liu R., Gao L., Xia Y., Jiang Y. (2021). Diagnostic value of sonographic features in distinguishing malignant partially cystic thyroid nodules: A systematic review and meta-analysis.. Front. Endocrinol..

[29] Zhou T., Huang H., Dong H., Ni Z., Sun H., He T., Ma C. (2023). Ultrasound-Based risk stratification system for the assessment of partially cystic thyroid nodules.. Endocr. Pract..

[30] Chiu F.Y., Yen Y. (2023). Imaging biomarkers for clinical applications in neuro-oncology: Current status and future perspectives.. Biomark. Res..

[31] Kurti M., Sabeti S., Robinson K.A., Scalise L., Larson N.B., Fatemi M., Alizad A. (2023). Quantitative biomarkers derived from a novel contrast-free ultrasound high-definition microvessel imaging for distinguishing thyroid nodules.. Cancers.

[32] Li Y., Li X., Yan L., Xiao J., Yang Z., Zhang M., Luo Y. (2025). Multiparametric ultrasound techniques are superior to AI-assisted ultrasound for assessment of solid thyroid nodules: A prospective study.. Endocrine.

[33] Xu S., Ni X., Zhou W., Zhan W., Zhang H. (2022). Development and validation of a novel diagnostic tool for predicting the malignancy probability of thyroid nodules: A retrospective study based on clinical, B-mode, color doppler and elastographic ultrasonographic characteristics.. Front. Endocrinol..

[34] Abbasian Ardakani A., Airom O., Khorshidi H., Bureau N.J., Salvi M., Molinari F., Acharya U.R. (2024). Interpretation of artificial intelligence models in healthcare.. J. Ultrasound Med..

[35] Zhao Y., Qi Y., Liu X., Cui Y., Zhao Z. (2022). Efficiency of disease and disease activity diagnosis models of systemic lupus erythematosus based on protein array analysis.. J. Immunol. Res..

[36] Chen Q., Liu Y., Liu J., Su Y., Qian L., Hu X. (2023). Development and validation of a dynamic nomogram based on conventional ultrasound and contrast-enhanced ultrasound for stratifying the risk of central lymph node metastasis in papillary thyroid carcinoma preoperatively.. Front. Endocrinol..

[37] St Fleur R.G., Ream M., Walsh E.A., Antoni M.H. (2025). Cognitive behavioral stress management affects different dimensions of benefit finding in breast cancer survivors: A multilevel mediation model.. Psychol. Health.

